# Progress in the application of traditional Chinese medicine and natural products for the prevention and treatment of calf diarrhea: a review

**DOI:** 10.3389/fvets.2026.1820966

**Published:** 2026-06-17

**Authors:** Huzaifa Iftikhar, Sai Wei, Zhe Wang, Zhenghan Chen, Long-ping Li, Tai-fei Bi, Lei Qu, Shu-wei Dong

**Affiliations:** Shaanxi Provincial Engineering and Technology Research Center of Cashmere Goats, College of Modern Agriculture, Yulin University, Yulin, China

**Keywords:** antibacterial, calf diarrhea, immunomodulation, natural products, plant extracts, traditional Chinese medicine

## Abstract

Calf diarrhea is a common and severe disease in the livestock industry, leading to poor growth and high mortality rates in calves, thereby causing significant economic losses. With increasing concerns over antibiotic resistance, traditional Chinese medicine (TCM) and natural products have garnered widespread attention as alternative therapeutic options for managing calf diarrhea. This review systematically summarizes recent advances in the utilization of TCM, natural products, and plant extracts for the prevention and treatment of calf diarrhea. The mechanisms underlying their antibacterial, anti-inflammatory, immunomodulatory, and intestinal protective effects are critically analyzed in this review. Additionally, the safety profiles and potential applications of these natural agents are evaluated to provide a comprehensive understanding of their roles in the control of diseases. By integrating the latest research findings, this review aims to offer theoretical support and practical guidance for the green and sustainable prevention and control of calf diarrhea, addressing the urgent need for effective, safe, and environmentally friendly therapeutic strategies in modern animal husbandry.

## 1 Introduction

Calf diarrhea is a widespread health problem in the cattle industry, particularly affecting neonatal calves whose gastrointestinal barriers and immune systems are not fully mature. This vulnerability makes newborn calves highly susceptible to enteric pathogens, often leading to diarrhea that causes significant morbidity and economic losses (1). Epidemiological data show that diarrhea is more prevalent during the suckling period than after weaning (2). The etiology of calf diarrhea is multifactorial, involving bacterial (e.g., *E. coli*, *Salmonella* spp.) and viral (e.g., bovine rotavirus) pathogens, many of which are emerging multidrug-resistant (MDR) strains. For example, *E. coli* isolates from diarrheic calves often resist multiple antibiotic classes and commonly carry genes like *bla*^TEM^ and *tetA* (3–5). These MDR strains frequently carry virulence factors (adhesins, toxins, and iron-scavenging systems) that enhance their pathogenicity (6, 7). The rise of such MDR pathogens threatens animal health and poses potential public health risks (8). Moreover, extensive antibiotic use in calf rearing has resulted in residues in animal products and disrupted the gut microbiota of calves, which is critical for their immune function and growth (9). Together, these factors underscore the need for alternative strategies in preventing and managing calf diarrhea.

Traditional management of calf diarrhea relies mainly on antibiotics and supportive care. However, widespread and indiscriminate antibiotic use has accelerated the emergence of resistant strains (3). For instance, *E. coli* and *Salmonella* isolated from diarrheic calves frequently show resistance to drugs such as amoxicillin, cefuroxime, and erythromycin (4). Resistance genes (e.g., *bla*^TEM^) are often carried on mobile genetic elements, facilitating their spread (3, 7). Antibiotics are also ineffective against viral or protozoan pathogens and can leave residues in food products (9), highlighting the limitations of antibiotic therapy and the need for alternatives. Immunotherapies, vaccines, and probiotics have been explored to improve calf health (6), but traditional Chinese medicines and other natural products have garnered interest as potentially safer alternatives for preventing calf diarrhea.

In recent years, traditional Chinese medicine (TCM) formulas and natural products have drawn attention as alternative treatments for calf diarrhea. These therapies often have fewer side effects, lower costs, minimal drug residues, and reduced risk of inducing resistance (10). For example, Cangpu Oral Liquid (CP), a modified herbal formula, demonstrated superior efficacy in neonatal calves with diarrhea compared to antibiotics, with higher recovery rates, shorter illness duration, and weight gain (10). Similarly, Pueraria polysaccharide (PPL), a natural compound with immunomodulatory and antioxidative properties was shown to restore health in diarrheic calves by improving liver function and gut barrier integrity (11). Another TCM formulation, Yigong San (YGS), alleviated diarrhea by enhancing antioxidant defenses, reducing pro-inflammatory cytokines, and modulating arginine and proline metabolism, thereby improving immune responses and metabolic profiles in affected calves (12). Additionally, probiotics (e.g., *Limosilactobacillus reuteri* and *Pediococcus pentosaceus* strains) have shown promise in preventing and treating calf diarrhea (13, 14). This review aims to summarize recent research progress on Chinese herbal medicines and natural products in preventing and treating calf diarrhea, providing a scientific basis for their broader adoption in animal husbandry. Throughout this review, we distinguish between evidence derived from *in vitro* experiments, animal models, and clinical studies in calves, as the translational relevance of each level differs substantially.

The literature search for this review was conducted using PubMed, and Google Scholar databases. The following keywords were used alone or in combination: *calf diarrhea*, *neonatal calf diarrhea*, *traditional Chinese medicine*, *natural products*, *plant extracts*, *antibacterial*, *anti-inflammatory*, *immunomodulation*, *gut microbiota*, and *intestinal barrier*. The search was primarily limited to articles published between 2015 and 2026, with older seminal studies included where relevant. Only peer-reviewed articles published in English were considered.

## 2 Etiology and pathogenesis of calf diarrhea

### 2.1 Major pathogenic microorganisms

Calf diarrhea usually arises from a combination of pathogens – viruses, bacteria, and parasites – acting together. The major infectious agents are:

*Viruses*: *bovine rotavirus* (BRV) and *bovine coronavirus* (BCoV) are the leading viral causes worldwide (15, 16). These pathogens infect the intestinal lining of newborn calves, causing malabsorption and secretory diarrhea. For example, a Korean study found rotavirus (BoRVA) mainly in calves under 30 days old, while a unique strain of BCoV was also detected (15). In China, rotavirus is similarly the dominant viral pathogen (16). Other emerging viruses – bovine kobuvirus (BKoV), astrovirus (BoAstV), and torovirus (BToV) – have been identified in diarrheic calves, though their exact role is still being studied (17). Multiplex RT-qPCR tests now allow rapid detection of these various viruses (17).

*Bacteria*: *Escherichia coli* (especially enterotoxigenic *E. coli* K99) and *Salmonella* spp. are key bacterial culprits. *E. coli* K99 colonizes the small intestine using fimbriae and secretes toxins that disrupt fluid absorption, leading to profuse diarrhea (18). Research shows that interventions like camel colostrum can inhibit pathogenic *E. coli* (e.g., strains with F17 adhesin) by reducing their growth and biofilm formation (18). In Ningxia, China, a combination of a Chinese herbal formula (CHF) and Microcin J25 acted synergistically to kill multidrug-resistant *E. coli* and *Salmonella* from calves, lowering diarrhea incidence and helping restore normal gut flora (19). Notably, some *E. coli* strains carry the high-pathogenicity island (HPI) and can trigger strong inflammation (via the TGF-β1/Smad3 pathway) in hosts, causing organ damage in experimental models (20). Antibiotic resistance among these bacteria is rising, which limits treatment and underlines the potential of alternatives like probiotics to support gut immunity (18–20).

*Parasites*: protozoa also play a major role. *Cryptosporidium parvum* is a leading protozoal cause, it invades intestinal cells, blunts the villi, and causes malabsorptive diarrhea. In some regions (e.g., Ningxia, China) over 50% of diarrheic calves tested positive for *Cryptosporidium* (16). Other parasites like *Giardia* spp. and *Eimeria* spp. (coccidia) contribute too (21). *Eimeria* infections often vary with calf age. Treatment is difficult: only a few drugs (e.g., diclazuril, toltrazuril) exist for coccidia (22). These parasitic infections can co-occur with bacteria and viruses, complicating disease control. Because protozoal diseases are hard to treat, maintaining a healthy gut microbiome is promising studies suggest that restoring microbial balance (for example via probiotics) could help prevent or reduce diarrhea severity (23).

These findings show that calf diarrhea is multifactorial. The mix of pathogens can vary by farm, region, and husbandry practices, so diagnostics must be broad. Modern tools like multiplex PCR and metagenomic sequencing are used to identify the spectrum of viral and bacterial agents (24, 25). Importantly, mixed infections are common. For example, calves co-infected with rotavirus and *Cryptosporidium* have higher morbidity and mortality (26), though the exact synergy is still under study. During diarrhea, the gut microbiota also changes dynamically – often with an expansion of Enterobacteriaceae and their phages (27) – which can set the stage for further infection. These insights suggest that maintaining *microbial homeostasis* (e.g., with probiotics or prebiotics) could improve outcomes (see Tables 1, 2, 3).

**Table 1:** The evidence of hierarchy.

Intervention	Evidence level	Model/subject	Key finding
Cangpu oral liquid	Clinical	246 Holstein calves (RCT)	Higher recovery rate, shorter illness vs. apramycin
Yigong san (YGS)	Clinical	Diarrheic Holstein calves	Reduced cytokines, improved antioxidant status
SBWP	Clinical	Simmental × Yili calves	Reduced diarrhea rate, improved growth
Gallic acid	Animal	Neonatal mouse model	Gut microbiota modulation, reduced colitis
Astragaloside IV	Animal	Immunosuppressed mice	Restored immune organ function
Radix Bupleuri polysaccharides	Animal	Immunosuppressed mice	Restored intestinal immune function
Berberine	In vitro	MRSA cell culture	Inhibits cell wall synthesis via tarO
Baicalin	In vitro	Bacterial cultures	Antibacterial via membrane disruption
Palmatine	In vitro	*E. coli* cultures	Restores ciprofloxacin sensitivity

**Table 2:** Summary of key TCM compounds and natural products reviewed.

Compound/product	Source/origin	Category	Mechanisms of action	Key evidence	Manuscript section
Baicalin/baicalein	*Scutellaria baicalensis*	Flavonoid	Cell wall disruption; inhibits nucleic acid synthesis; quorum sensing interference; synergy with meropenem/polymyxin E	Enhanced antibacterial activity vs. MDR *Klebsiella pneumoniae*; biofilm eradication via LuxS/LuxR downregulation	§3.1, §3.3
Berberine	Coptis chinensis	Alkaloid (Protoberberine)	Inhibits tarO gene (cell wall teichoic acid biosynthesis); upregulates autolytic enzymes LytM/SsaA; self-assembles with rhein into nanoparticles	Potent activity vs. MRSA; synergy with oxacillin; resistance reversal demonstrated in vitro	§3.1, §3.3
Gallic acid (GA)	Multiple plant sources; polyphenol	Polyphenol monomer	Inhibits bacterial growth & adherence; modulates NF-κB/MAPK; enriches SCFA-producing microbiota (Clostridia, Lachnospiraceae)	Reduced colonic inflammation vs. ESBL-EAEC in neonatal calves; FMT confirmation of microbiota transfer	§4.2, §6.1, §8.2
Matrine/*Isatis tinctoria* alkaloids	*Sophora flavescens*; *Isatis tinctoria*	Alkaloid	Targets bacterial DNA replication/protein synthesis; modulates resistance mechanisms; broad-spectrum	Antibacterial activity vs. Gram-positive and Gram-negative pathogens; membrane disruption demonstrated	§3.1
Yigong San (YGS)	Multi-herb TCM formula	Compound TCM formulation	Suppresses TNF-α, IL-1β, IL-17A, IFN-γ; upregulates SOD, CAT, GPx; modulates arginine/proline metabolism; NF-κB inhibition	RCT in 20 diarrheic Holstein calves; significant improvement in biochemical markers; metabolomic profiling confirmed mechanism	§4.1, §5.2, §6.3, §7.1, §8.1
Cangpu Oral Liquid (CP)	Modified traditional herbal formula (Cang-ai volatile oil base)	Compound TCM formulation	Multi-target antibacterial and immunomodulatory; reduces inflammation and supports gut repair	Multicenter RCT (*n* = 246): 82.1% vs. 62.6% recovery rate vs. apramycin; shorter recovery (3.9 vs. 6.6 days); higher ADG	§8.1, §8.3
SBWP (*Saccharomyces boulardii* cell wall polysaccharide)	*Saccharomyces boulardii* (yeast)	Prebiotic/polysaccharide	NF-κB inhibition; increases IL-10; reduces IL-1β, IL-6, TNF-α; enriches Lactobacillus/Bifidobacterium; reduces pathogenic coliforms	500 mg/day in calves: −31.6% fecal score, −18.5% diarrhea rate, +28.5% ADG vs. control	§4.2, §4.3, §5.3, §8.2
Pueraria Polysaccharide (PPL)	*Pueraria lobata* (kudzu root)	Polysaccharide	Immunomodulatory; antioxidative; restores liver function and gut barrier integrity	Restored health in diarrheic calves; improved liver function markers via blood metabolomics	§1
Astragaloside IV (AS-IV)	*Astragalus mongholicus*	Saponin	Activates HIF-1α/NF-κB; enhances macrophage migration/phagocytosis; restores NK cell activity; improves lymphocyte proliferation	Restored immune organ indices in immunosuppressed mouse models; significant enhancement of innate immunity	§5.1
Zinc (Zn) supplementation	Micronutrient; inorganic/organic forms	Micronutrient	Upregulates A20 (negative regulator of NF-κB); improves tight junction expression; increases IgG/IgM production; reduces pro-inflammatory cytokines	Scoping review: improved epithelial barrier; increased immunoglobulins; shorter illness duration; evidence rated moderate	§4.2, §5.3, §8.1
*Pediococcus pentosaceus* SNF15	Probiotic (lactic acid bacteria)	Probiotic	Upregulates occludin, claudin, ZO-1, MUC2; reduces pro-inflammatory cytokines; TLR-mediated immune activation	Improved intestinal mucosal integrity in murine *E. coli* K99 diarrhea model; whole genome sequencing confirmed safety	§5.3
Radix bupleuri polysaccharides (RBP)	Bupleurum Chinense	Polysaccharide	Activates TLR2/TLR4 → MAPK/NF-κB; enhances macrophage phagocytosis; modulates CD4 + T cell differentiation; increases SCFA production	Restored intestinal immune function in cyclophosphamide-immunosuppressed mice; microbiota normalization confirmed	§5.1

ADG, average daily gain; MRSA, methicillin-resistant *Staphylococcus aureus*; ESBL-EAEC, extended-spectrum β-lactamase-producing enteroaggregative *E. coli*; SCFA, short-chain fatty acid; SOD, superoxide dismutase; CAT, catalase; GPx, glutathione peroxidase; FMT, fecal microbiota transplantation.

**Table 3:** Comparison of key clinical and experimental studies.

Intervention	Study design	Population	Primary outcomes	Key strengths	Limitations
Cangpu oral liquid vs apramycin	Randomized controlled field trial	246 diarrheic Holstein calves, aged 2–15 days	Recovery rate: 82.1% (CP) vs. 62.6% (ABX); recovery time: 3.9 vs. 6.6 days (*P* = 0.001); ADG: 211.45 vs. 164.56 g/day (*P* = 0.001)	Large sample size; active comparator; clinical relevance; ADG as economic endpoint	Single center; no pathogen stratification; no microbiota data; no mechanistic analysis
Yigong San (YGS)	Controlled observational study	20 diarrheic Holstein calves + healthy controls; 7-day treatment	Reduced TNF-α, IL-1β, IL-17A; increased SOD, CAT, GPx; decreased MDA; metabolomic pathway enrichment (arginine/proline)	Metabolomic profiling; multi-cytokine panel; mechanistic depth; antioxidant and immune endpoints	Small sample (*n* = 20); no antibiotic comparator; 7-day follow-up only; no pathogen identification
SBWP supplementation	Randomized trial	Simmental × Yili brown calves; 500 mg/day supplementation	Fecal score −31.6%; diarrhea rate −18.5%; ADG + 28.5%; feed-to-gain ratio −23%; IgG increased; TNF-α/IL-1/IL-6 reduced	Multiple endpoints (growth, immunity, microbiota); clear dose specification; practical supplementation protocol	Single breed; no pathogen confirmation; no antibiotic comparator; short duration
Gallic acid (GA) vs ESBL-EAEC	Controlled experimental study + FMT validation	Neonatal dairy calves and neonatal mice (*E. coli* challenge model)	Reduced colonic inflammation; restored hindgut SCFAs; enriched Clostridia/Lachnospiraceae; FMT conferred protection	FMT validation strengthens causal inference; multi-species confirmation; microbiota mechanism elucidated	Mouse model for FMT component; limited calf-specific clinical outcome data; no field application data
Pueraria polysaccharide (PPL)	Metabolomics-based clinical study	Diarrheic calves; blood metabolomics assessment	Restored health; improved liver function; improved gut barrier integrity confirmed by metabolomic profiling	Blood metabolomics provides systemic view; demonstrates hepato-protective effect	Mechanistic details limited; small cohort; no randomization described; single center
IgY DNT passive immunization	Clinical trial in newborn Holstein calves	Newborn Holstein calves; twice-daily oral IgY for first 2 weeks of life	Delayed diarrhea onset; reduced severity and duration; shortened viral shedding period vs. untreated controls	Targets multiple pathogens (rotavirus, coronavirus, ETEC, Salmonella); biological alternative; neonatal window strategy	No antibiotic comparator; limited data on microbiota effects; cost and scalability not assessed
Zinc (Zn) supplementation	Scoping review of multiple studies	Multiple calf breeds and age groups across reviewed studies	Moderate evidence for improved epithelial barrier; increased IgG/IgM; reduced pro-inflammatory cytokines; shorter illness duration	Broad evidence synthesis; regulatory-grade safety profile; practical micronutrient application	Heterogeneous study designs; evidence rated ‘moderate’; dosing not standardized; T-cell/innate cell effects inconclusive

In practice, an integrated approach is recommended. This includes vaccination (e.g., against rotavirus, coronavirus, and K99 *E. coli*), good colostrum management (high-immunity transition milk), targeted antibiotic use, and alternative therapies (28). Together, vaccination and passive immunity have been shown to reduce calf diarrhea mortality (28). Combining improved hygiene, nutrition, and novel treatments (like herbal remedies or probiotics) is therefore essential to mitigate the burden of calf diarrhea in livestock operations.

### 2.2 Immune system dysregulation and intestinal barrier disruption

Neonatal calves have an immature immune system that cannot fully defend against gut pathogens. Key factors in this vulnerability include:

Immune checkpoints: Inflammation in calves upregulates inhibitory molecules. For example, high PD-L1 expressions on calf macrophages (as seen in severe sepsis models) can suppress T-cell function (29). This checkpoint-mediated immunosuppression likely occurs in diarrheic calves too, reducing their ability to clear infection.

*Lymphocyte loss*: inflammatory states cause T-cell counts to drop and splenocyte apoptosis to rise, further weakening systemic immunity. A lack of sufficient T cells makes it easier for gut pathogens to establish and spread.

*Barrier breakdown*: the intestinal mucosa normally blocks pathogens, but inflammation damages this barrier. Tight junction proteins become disorganized, enterocytes undergo apoptosis, and mucosal inflammation sets in. As a result, bacteria and toxins leak into tissues, triggering even more inflammation.

*Myeloperoxidase (MPO)*: neutrophils release MPO, an enzyme with a dual role. Under normal conditions MPO helps kill microbes and modulates oxidative stress, but if uncontrolled, MPO itself can intensify tissue oxidation and injury (30). Natural antioxidants (e.g., from herbs) have been shown to regulate MPO activity, suggesting a potential therapy to protect the gut barrier (30).

*COX-2 and inflammation*: inflammatory mediators like COX-2 become upregulated (studied in skin but relevant to gut). COX-2 drives prostaglandin production and amplifies local inflammation (31). Phytochemicals that inhibit COX-2 (with good safety profiles) could therefore help reduce gut inflammation and support healing (31).

*Gut microbiome*: early colonization by beneficial microbes is critical for immune maturation. Disruption of these commensal communities is closely linked to diarrhea: a healthy microbiome helps maintain barrier function and resist pathogens (32). Thus, interventions that restore a balanced microbiome (e.g., probiotics, prebiotics, herbal modulators) may synergize with immune therapies to improve gut integrity (29–32).

In summary, immune imbalance and barrier loss create a vicious cycle: weakened immunity allows pathogens to invade the gut, and barrier damage fuels more inflammation. Effective strategies should therefore target both immunity and barrier integrity. For example, combining immune checkpoint inhibitors, antioxidants, and microbiota-based therapies could strengthen calves’ resistance (29–32). The fact that many traditional herbal compounds modulate MPO, PD-L1, and COX-2 supports exploring these natural products for diarrhea prevention (29–32).

### 2.3 Role of inflammatory response and oxidative stress in diarrhea

Inflammation and oxidative stress are central to how diarrhea damages the gut:

*Pro-inflammatory cytokines*: diarrheic calves exhibit elevated levels of TNF-*α*, IL-1β, IL-6, IL-8, and IFN-*γ* in blood and gut tissue (33–36). Although piglet models of *E. coli* infection showed mixed results on direct cytokine correlation (33, 34), many studies find calves with diarrhea have high systemic TNF-*α* and other cytokines, indicating a strong inflammatory state (35, 36). This cytokine storm injures the mucosa – cells undergo atrophy and villus flattening – impairing nutrient absorption and promoting fluid loss (37, 38).

**Figure 1:**
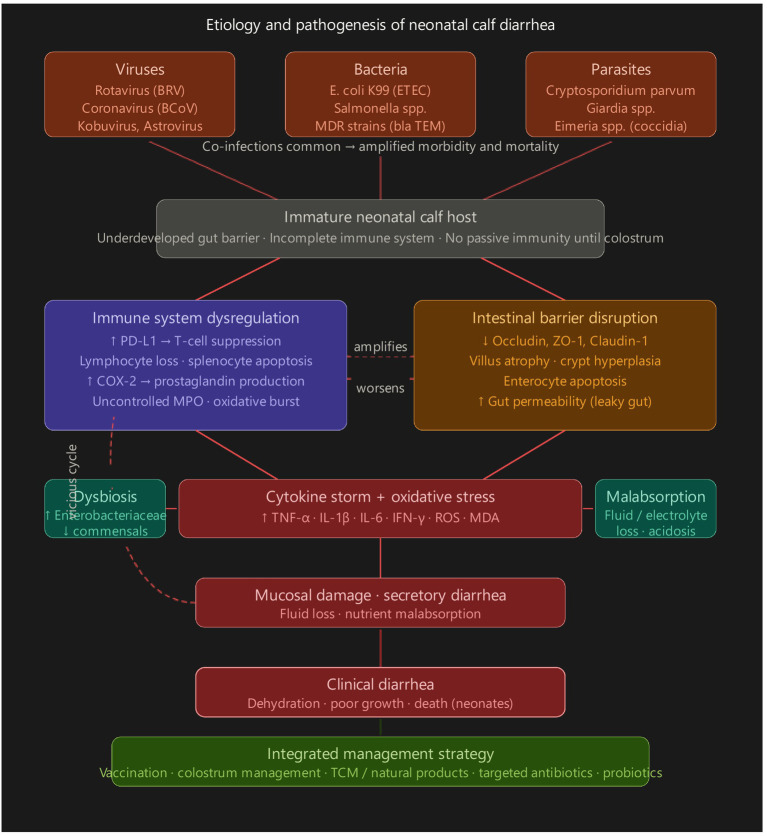
Etiology and pathogenesis of neonatal calf diarrhea.

*NF-κB signaling*: pathogens trigger NF-κB activation in the intestinal mucosa (39, 40). NF-κB then drives further cytokine production, creating a feedback loop. This amplifies mucosal damage: increased crypt cell proliferation, villus destruction, and increased gut permeability (37–40) (Figure 1).

*Oxidative stress*: diarrhea causes an imbalance between reactive oxygen species (ROS) and the gut’s antioxidant defenses (41, 42). Markers like malondialdehyde (MDA) and 8-OHdG often rise, though calf studies show inconsistent results (41, 42). Excess ROS damages enterocytes, causing apoptosis, and degrades tight junction proteins (e.g., occludin, ZO-1) (43, 44). This increases leakiness of the gut lining and exacerbates diarrhea (43, 44).

*Antioxidant enzymes*: key enzymes (superoxide dismutase, catalase, glutathione peroxidase) are typically suppressed in diarrhea, although results vary (36, 45). Supplementing antioxidants has shown promise: for instance, curcumin or herbal polysaccharides given to animals with induced diarrhea restored these enzymes’ activity, lowered oxidative markers, and improved intestinal barrier function (46–48).

*Crosstalk between inflammation and oxidation*: immune cells naturally produce ROS to kill pathogens (49). However, excess ROS also triggers inflammatory pathways (50, 51), making the processes self-reinforcing. Clinical observations support this interplay: for example, in pediatric COVID-19 patients, diarrhea correlated with higher C-reactive protein and cytokines (40, 52). Chemokines like CXCL10 and CXCL11 are elevated as well, drawing more CD4 + T cells into the gut and perpetuating inflammation (40).

**Figure 2:**
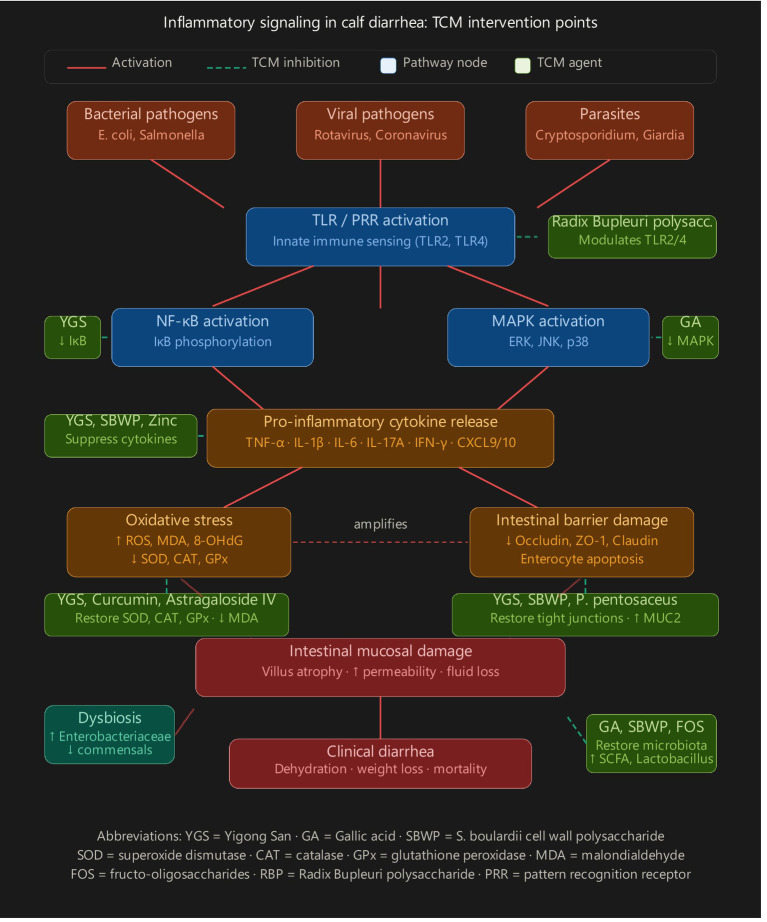
The inflammatory pathway (Inflammatory signaling in calf diarrhea: TCM intervention points).

This inflammation–oxidation loop perpetuates tissue damage and fluid loss. Breaking the cycle is a therapeutic goal. Anti-inflammatory agents (including non-steroidal anti-inflammatories and herbal COX-2 inhibitors) and antioxidants (from herbs or nutraceuticals) have shown efficacy in reducing diarrhea symptoms (12, 39, 53, 54). Indeed, many traditional Chinese medicine (TCM) formulas that alleviate calf diarrhea work by lowering cytokines and oxidative stress (12, 53, 54). Future research should delve into the precise molecular interactions in these pathways. Understanding exactly how specific compounds influence NF-κB, ROS, or mucosal healing will help optimize treatments and minimize diarrhea in neonatal calves (see Figure 2).

## 3 Antibacterial effects of traditional Chinese medicine and natural products

### 3.1 Typical antibacterial activities of Chinese herbal components

Traditional Chinese medicine has long been recognized for its rich repository of bioactive compounds with significant antibacterial properties, particularly flavonoids and alkaloids. Among these, flavonoids such as berberine and baicalin and alkaloids including matrine and compounds derived from *Isatis tinctoria* have been extensively studied for their efficacy against various bacterial pathogens, including multidrug-resistant strains.

**Figure 3:**
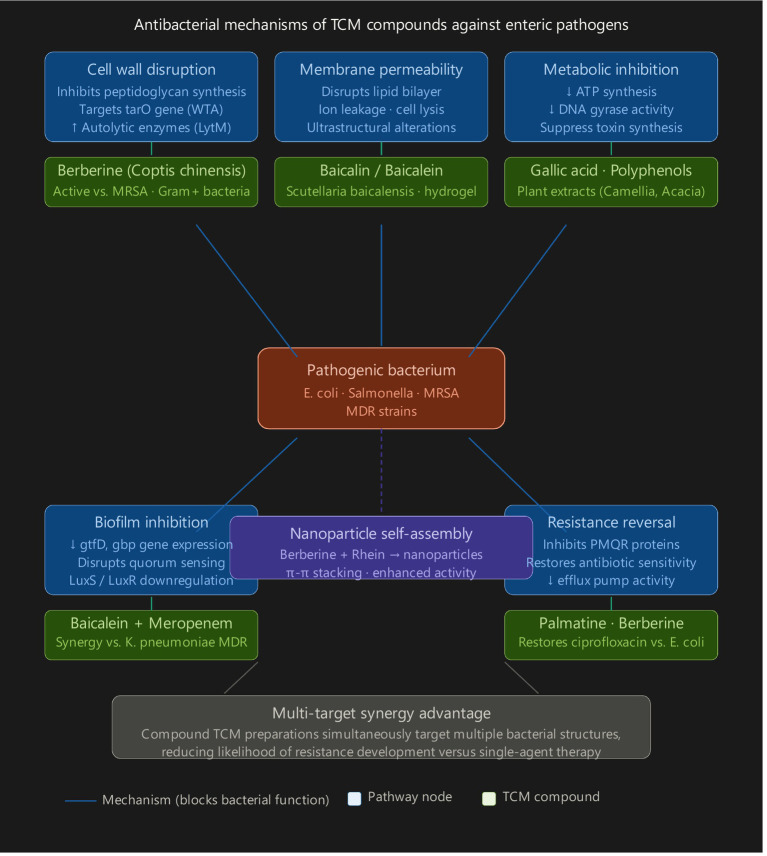
Antibacterial mechanisms of TCM compounds against enteric pathogens.

Flavonoids, a large class of polyphenolic compounds, are prominent in many heat-clearing TCM herbs and exhibit diverse antibacterial activities. For instance, baicalin, a flavone glycoside predominantly found in *Scutellaria baicalensis*, demonstrates potent antibacterial activity. However, its clinical application is limited by its poor solubility. Recent advances in formulation, such as the development of dynamic covalent hydrogels composed solely of baicalin and inorganic borate, have improved their solubility and antibacterial efficacy (55). These hydrogels exhibit remarkable thixotropy, self-healing, and moldability, providing a promising drug delivery system that improves the therapeutic potential of baicalin. Furthermore, flavonoids exert antibacterial effects through multiple mechanisms, including disruption of bacterial membranes, inhibition of nucleic acid synthesis, and interference with bacterial energy metabolism. Their ability to act as direct-acting antibacterial compounds (DACs) and host-acting antibacterial compounds (HACs) highlight their versatility in combating resistant bacteria (56). Notably, the structural features of flavonoids, such as hydroxyl groups and conjugated double bonds, are critical for their antibacterial activity, facilitating interactions with bacterial proteins and membranes (Figure 3).

Alkaloids, particularly berberine, have attracted considerable attention because of their broad-spectrum antibacterial properties. Berberine, a protoberberine alkaloid found in *Coptis chinensis* and related plants, exhibits potent activity against gram-positive bacteria, including methicillin-resistant *Staphylococcus aureus* (MRSA) (57). *In vitro* studies show that berberine disrupts bacterial cell wall synthesis by inhibiting the tarO gene, a key enzyme involved in wall teichoic acid biosynthesis, thereby compromising cell wall integrity and promoting bacterial lysis. Moreover, berberine upregulates bacterial autolytic enzymes, such as LytM and SsaA, further enhancing bacterial cell wall degradation (57). Notably, berberine can self-assemble with other phytochemicals, such as rhein, to form nanoparticles with enhanced antibacterial activity against MRSA (58, 59). This self-assembly involves hydrogen bonding and *π*–*π* stacking, which stabilizes the nanoparticles and facilitates their interaction with bacterial surfaces, leading to effective bacterial inhibition.

Other alkaloid-containing TCM components, such as matrine and compounds from *Isatis tinctoria* and *Phellodendron chinense*, also exhibit antibacterial properties. These alkaloids often target bacterial DNA replication or protein synthesis and modulate bacterial resistance mechanisms. For example, the ethyl acetate extract of *Taxillus chinensis* contains 4-indolecarbaldehyde, which exhibits broad-spectrum antibacterial activity by disrupting bacterial membrane permeability, causing morphological abnormalities, and increasing bacterial death (60). This highlights the importance of alkaloids in targeting bacterial membranes and their potential to overcome bacterial resistance mechanisms.

Flavonoids and alkaloids also exhibit synergistic effects when combined with conventional antibiotics, reducing the required dosage and mitigating the development of resistance (61). The multifaceted antibacterial mechanisms of these compounds, including the inhibition of biofilm formation, interference with quorum sensing, and modulation of bacterial metabolism, make them promising candidates for adjunct therapies for bacterial infections. Moreover, their natural origin and lower likelihood of inducing resistance confer an advantage over synthetic antibiotics.

In conclusion, typical antibacterial components of TCM, such as flavonoids (e.g., baicalin and baicalein) and alkaloids (e.g., berberine and 4-indolecarbaldehyde) exhibit potent antibacterial activities through diverse mechanisms including cell wall disruption, membrane permeability alteration, and interference with bacterial metabolism. Their ability to self-assemble into nanoparticles and synergize with antibiotics further enhances their therapeutic potential. Continued research on these compounds, including optimization of delivery systems and mechanistic elucidation, will advance their application in preventing and treating bacterial infections (55, 57, 58, 60).

### 3.2 Mechanisms of plant extracts in inhibiting pathogenic Bacteria

Plant extracts have garnered considerable attention as alternative antimicrobial agents owing to their multifaceted mechanisms against pathogenic bacteria, which are particularly relevant in the prevention and treatment of calf diarrhea caused by bacterial infections. One primary mechanism by which plant extracts exert antibacterial effects is through the disruption of bacterial cell wall integrity. The bacterial cell wall, composed mainly of peptidoglycan in gram-positive bacteria and an outer membrane with lipopolysaccharides in gram-negative bacteria, is essential for maintaining cell shape and protecting against environmental stress. Polyphenol-rich plant extracts, such as those derived from *Camellia sinensis* and *Acanthaceae* family plants, have been shown to induce morphological changes in bacteria, leading to cell wall damage and increased bacterial permeability. This disruption compromises bacterial viability by damaging the cell wall and metabolic processes (62, 63). For instance, *in vitro*, methanolic extracts of *Cinnamum zeylanicum* and *Acacia nilotica* demonstrated significant antibacterial activity against *Staphylococcus aureus* and *Streptococcus pyogenes* (64). Similarly, the brown alga *Sargassum muticum* extract caused ultrastructural alterations, such as cell wall thickening and cytoplasmic disintegration, in *Fusarium moniliforme*, indicating that plant-derived compounds can target cell envelope components of diverse pathogens (65). These findings underscore the potential of plant extracts to directly compromise bacterial cell wall integrity, which is a fundamental antibacterial mechanism.

In addition to physically disrupting the cell wall, plant extracts inhibit bacterial metabolism and toxin production, which are critical for pathogen survival and virulence. Bioactive compounds, such as flavonoids, alkaloids, and tannins, interfere with bacterial enzymatic activity and metabolic pathways. For example, extracts from *Suaeda aegyptiaca*, which contain alkaloids and flavonoids, have been shown to potently inhibit both Gram-positive and Gram-negative bacteria (66). Moreover, polyphenols in plant extracts can suppress ATP synthesis and DNA gyrase activity, thereby hindering bacterial energy production and DNA replication (62). Inhibition of toxin synthesis is particularly relevant in pathogens causing calf diarrhea, such as enteroaggregative *Escherichia coli*. Gallic acid, a plant-derived secondary metabolite, has been demonstrated to reduce bacterial growth and adherence and diminish lipopolysaccharide-induced inflammation in intestinal epithelial cells, suggesting a dual role in metabolic inhibition and attenuation of bacterial virulence (67). Additionally, bio converted milk containing *Artemisia herba-alba* extracts exhibited antimicrobial effects against periodontal pathogens by inhibiting bacterial growth and enzymatic activities related to virulence (68). The suppression of bacterial metabolism and toxin production by plant extracts not only reduces bacterial proliferation but also mitigates the pathogenic effects on the host.

Furthermore, plant extracts can interfere with bacterial biofilm formation, which is a key factor in persistent infections and antibiotic resistance. *Acanthaceae* family plant extracts inhibit biofilm-related genes, such as gtfD and gbp, which facilitate bacterial adherence and biofilm maturation (63). By preventing biofilm formation, plant extracts enhance bacterial susceptibility to immune clearance and antimicrobial agent. This mechanism complements the direct antibacterial effects on the cell wall and metabolism, contributing to a comprehensive antibacterial strategy. The combination of these mechanisms may explain the broad-spectrum antibacterial efficacy observed with various plant extracts, including those from *Vernonia auriculifera* and *Buddleja polystachya*, which exhibit greater antibacterial activity than gentamicin against selected pathogens (69).

The antibacterial efficacy of plant extracts is influenced by their phytochemical composition, extraction methods, and the target bacterial species. Methanol is a commonly used solvent for extracting bioactive compounds such as flavonoids, alkaloids, saponins, sterols/triterpenes, and tannins, which are responsible for antibacterial activities (66, 69). Notably, plant extracts tend to exhibit stronger activities against gram-positive bacteria (70). However, some extracts also showed significant inhibition of gram-negative bacteria, indicating a broad spectrum of action (71). The presence of multiple bioactive compounds within a single extract may have synergistic effects, enhance antibacterial potency and reducing the likelihood of resistance development. For example, the synergistic antibacterial activity of *Vernonia auriculifera* and *Buddleja polystachya* extracts was greater than that of the individual extracts, suggesting combinatorial effects on bacterial targets (69).

Collectively, the antibacterial mechanisms of plant extracts against pathogenic bacteria involve disruption of cell wall integrity, inhibition of bacterial metabolism and toxin production, and interference with biofilm formation. These multifaceted actions provide a promising basis for developing plant-based therapeutics to control bacterial infections in calves, particularly those that cause diarrhea. Given the rising concern over antibiotic resistance, plant extracts offer an alternative or complementary approach that may reduce the reliance on conventional antibiotics while preserving gut microbiota balance. Future research should continue to explore the antibacterial mechanisms of plant bioactive compounds and their applications (62, 67).

### 3.3 Synergistic antibacterial effects of compound traditional Chinese medicine preparations

Compound traditional Chinese medicine preparations, composed of multiple herbal components, exhibit enhanced antibacterial efficacy through synergistic interactions among their diverse bioactive constituents. This multi-component synergy allows for the simultaneous targeting of various bacterial structures and metabolic pathways, thereby improving overall therapeutic outcomes compared to single-agent treatments. For instance, core compounds such as baicalein, baicalin, forsythoside B, and forsythin, derived from classical TCM formulas, have demonstrated potent antibacterial activity against multidrug-resistant bacteria, including *Klebsiella pneumoniae* strains resistant to multiple antibiotics (72). These compounds not only inhibit bacterial growth but also disrupt biofilm formation, which is a critical factor in chronic and resistant infections. The combination of baicalein with conventional antibiotics, such as meropenem or polymyxin E, has been shown to produce synergistic effects, significantly enhancing bactericidal activity and biofilm eradication by interfering with bacterial quorum sensing systems through downregulation of the LuxS and LuxR genes (72). Such multi-target actions exemplify how TCM compounds can potentiate antibacterial efficacy beyond the sum of individual components.

Moreover, the synergistic effects of compound TCM formulations extend to reducing the risk of bacterial resistance. The complexity of these preparations, which contain numerous active ingredients with diverse mechanisms, makes it difficult for pathogens to simultaneously develop resistance against all components. For example, palmatine, an isoquinoline alkaloid from TCM, inhibits plasmid-mediated quinolone resistance proteins in *Escherichia coli*, restoring ciprofloxacin sensitivity by binding to resistance-associated proteins and reducing their protective effects (73). When used in combination with antibiotics, palmatine enhances antibacterial efficacy and reduces the selective pressure that typically drives the emergence of resistance. Similarly, berberine exhibits a synergistic effect with oxacillin against methicillin-resistant *Staphylococcus aureus* by compromising bacterial cell wall integrity through the inhibition of wall teichoic acid biosynthesis and upregulation of autolytic enzymes (57). These findings indicate that compound TCM preparations can act as resistance modulators, potentially reversing or preventing the development of antibiotic resistance in bacteria.

The multi-target and multi-pathway nature of compound TCM formulations also contributes to their broad-spectrum antibacterial activity and ability to interfere with bacterial virulence factors. For example, psoralen, derived from *Psoralea corylifolia*, inhibits quorum-sensing regulators in *Pseudomonas aeruginosa*, reducing virulence factor production and biofilm formation without exerting direct bactericidal effects, and shows synergistic antibacterial effects when combined with conventional antibiotics (74). This antivirulence strategy, facilitated by compound preparations, may reduce bacterial pathogenicity and improve host defense while minimizing the selective pressure for resistance. Furthermore, complex herbal mixtures, such as the Shensheng-Piwen changed medicinal powder (SPC) extract, have demonstrated significant antibiofilm activity and synergism with metal–organic frameworks, suggesting the potential for innovative combination therapies that enhance antibacterial efficacy and prevent resistance (75).

In addition to antibacterial synergy, compound TCM preparations often exhibit complementary pharmacological effects, such as anti-inflammatory and immune-modulating activities, which further support their use in infection control. For instance, extracts from *Liriodendron chinense* leaves contain multiple compounds that synergistically exert antioxidant, antimicrobial, and anti-inflammatory effects, helping alleviate infection-induced tissue damage and inflammation (76). The integration of such multifaceted actions in compound preparations may improve clinical outcomes by directly combating pathogens and modulating host responses.

Taken together, the evidence supports that compound traditional Chinese medicine preparations achieve superior antibacterial effects through the synergistic action of multiple bioactive constituents targeting diverse bacterial processes and their virulence factors. This multi-component synergy not only enhances efficacy but also reduces the likelihood of resistance development, offering a promising strategy for the prevention and treatment of infectious diseases, including those caused by multidrug-resistant pathogens. Future research should focus on elucidating the precise molecular interactions among the components and optimizing the formulations to maximize synergistic benefits while ensuring safety and clinical applicability.

## 4 Anti-inflammatory effects of traditional Chinese medicine and natural products

### 4.1 Inhibition of inflammatory cytokine expression

Inflammatory cytokines (TNF-*α*, IL-6, IL-1β) are critical drivers of calf diarrhea, which often involves intestinal inflammation and oxidative stress. Emerging evidence suggests that traditional Chinese medicine (TCM) formulations and natural products can modulate these mediators to alleviate diarrhea and improve calf health. For example, Yigong San (YGS) significantly suppresses multiple proinflammatory cytokines (TNF-*α*, IL-1β, IL-17A) in diarrheic calves; a controlled study found that 7 days of YGS treatment in Holstein calves markedly reduced serum TNF-α, IL-1β, IL-17A and the chemokines CXCL9 and CXCL10 (clinical study) (12). YGS also increased antioxidant enzymes (catalase, glutathione peroxidase, superoxide dismutase), lowered malondialdehyde, and shifted arginine/proline metabolism, indicating it both downregulates inflammation and restores oxidative balance (12).

Studies of neonatal calves with diarrhea show elevated serum and fecal reactive oxygen species (ROS), malondialdehyde and 8-hydroxy-2′-deoxyguanosine, along with decreased antioxidant enzyme activities (e.g., catalase). These oxidative changes align with increased serum TNF-*α*, IL-1β and IL-6. Importantly, calves recovering from diarrhea normalize both oxidative stress markers and cytokine levels, highlighting a tight interplay between redox balance and inflammation (41). This suggests that targeting oxidative stress could indirectly suppress inflammatory cytokines and aid intestinal recovery.

Natural products like YGS likely achieve cytokine suppression by restoring redox homeostasis and inhibiting NF-κB and other proinflammatory signaling pathways. Since excessive, sustained cytokine production exacerbates mucosal damage and diarrhea severity, their downregulation is therapeutically valuable. Notably, the observed negative correlation between ornithine (a metabolic intermediate) and proinflammatory cytokines suggests that metabolites may directly regulate inflammation (12). Overall, combining antioxidant enhancement with cytokine suppression offers an integrated approach that may provide a more effective, safer alternative to antibiotics in managing calf diarrhea.

### 4.2 Regulation of inflammatory signaling pathways

Controlling inflammation in calf diarrhea involves modulation of key pathways, notably NF-κB and MAPK. NF-κB drives expression of proinflammatory cytokines (IL-1β, IL-6, TNF-*α*) that rise during neonatal enteric infections (45). Natural compounds can attenuate this response. For example, *Saccharomyces boulardii* cell wall polysaccharide (SBWP) supplementation in newborn calves reduced IL-1β, IL-6, and TNF-α by 30.47%, 28.17%, and 25.49%, respectively, while increasing IL-10 by 45.45% (45), indicating NF-κB inhibition. SBWP-fed calves also showed lower diarrhea incidence and improved growth.

Gallic acid (GA), a plant polyphenol, similarly modulates NF-κB and MAPK pathways. In neonatal calves infected with ESBL-producing enteroaggregative *E. coli*, In a neonatal mouse model GA pretreatment reduced colonic inflammation and bacterial adherence (67). GA also restored hindgut short-chain fatty acids and enriched beneficial microbes (*Clostridia*, *Lachnospiraceae*) (67), which further supports anti-inflammatory effects through both direct pathway inhibition and SCFA-mediated regulation.

Micronutrients like zinc (Zn) contribute to pathway regulation. Zn supplementation decreases proinflammatory cytokines and enhances epithelial barrier function (77). Mechanistically, Zn upregulates A20, a negative regulator of NF-κB, reducing IL-1β and TNF-*α* production (77), while improving villus height and tight junction expression. These effects correlate with reduced diarrhea incidence, highlighting Zn’s role in controlling inflammation-driven disease.

Collectively, SBWP, GA, and Zn converge on NF-κB/MAPK inhibition, lowering proinflammatory cytokines, elevating IL-10, and restoring gut homeostasis (45, 67, 77). This integrated modulation of immune signaling and microbiota suggests potential as alternatives or complements to antibiotics, with added benefits for growth and feed efficiency. Further research is needed to define precise molecular targets and optimal dosing for therapeutic use in calf diarrhea.

### 4.3 Alleviating intestinal mucosal inflammatory injury [Analysis content]

The intestinal mucosa is a critical barrier against pathogen invasion and is essential for nutrient absorption and immune regulation. In neonatal calf diarrhea, mucosal inflammation and epithelial damage contribute significantly to disease progression, making mucosal repair an important therapeutic target. Natural products and traditional Chinese medicine components have shown potential in promoting mucosal healing through anti-inflammatory, immunomodulatory, and microbiota-regulating effects.

*Saccharomyces boulardii* cell wall polysaccharide (SBWP) has been reported to improve growth performance and reduce diarrhea incidence in newborn calves by modulating intestinal immunity and microbial composition (45). Calves supplemented with 500 mg/day SBWP exhibited increased serum IgG and IL-10 levels, alongside reductions in IL-1, IL-6, and TNF-*α* (45), indicating a shift toward an anti-inflammatory environment favorable for mucosal recovery. SBWP also increased beneficial bacterial populations, including Lactobacillus and Bifidobacterium, while reducing fecal *Escherichia coli* and *Clostridium perfringens* (45). These microbial changes are likely to contribute to epithelial regeneration and enhanced barrier integrity, supporting intestinal healing in diarrheic calves.

Similarly, Gallic acid (GA), a plant-derived polyphenol, has demonstrated efficacy in alleviating intestinal inflammation caused by extended-spectrum *β*-lactamase-producing enteroaggregative *Escherichia coli* infection (67). GA administration reduced colonic inflammation and restored short-chain fatty acid production, which is important for epithelial energy metabolism and mucosal regeneration. In addition, GA promoted the enrichment of beneficial taxa such as Clostridia_UCG-014 and *Lachnospiraceae* (67), suggesting that microbiota-derived metabolites contribute to its reparative effects. Fecal microbiota transplantation from GA-treated subjects further enhanced resistance to bacterial infection and inflammation, highlighting the interaction between GA, microbial balance, and mucosal healing.

The beneficial effects of SBWP and GA appear to involve suppression of inflammatory signaling, enhancement of anti-inflammatory cytokines, and restoration of epithelial barrier integrity. Improved microbial diversity and increased SCFA-producing bacteria may further support epithelial proliferation and differentiation. Because calf diarrhea involves both immune dysregulation and microbial imbalance, natural compounds targeting these interconnected mechanisms may provide synergistic benefits for mucosal regeneration.

Improved mucosal integrity may also explain the enhanced growth performance and feed efficiency observed in SBWP-supplemented calves (45), emphasizing the importance of intestinal repair in calf health and productivity. Further studies are needed to clarify the molecular pathways involved and to evaluate the efficacy of these compounds across different calf breeds and diarrheal etiologies.

In summary, natural products such as SBWP and GA demonstrate promising potential in reducing intestinal inflammation and promoting mucosal repair in neonatal calves. Their combined effects on immune regulation and microbiota composition support their application as alternatives or adjuncts to conventional therapies for calf diarrhea management. Continued investigation into their mechanisms of action and optimal dosing strategies will be important for clinical application.

## 5 Immunomodulatory effects of traditional Chinese medicine and natural products

### 5.1 Promotion of immune cell activity

The enhancement of immune cell function, particularly macrophages and lymphocytes, is an important mechanism by which traditional Chinese medicines and natural products protect against infectious and inflammatory diseases, including diarrhea in calves. Macrophages act as frontline innate immune cells through pathogen recognition, phagocytosis, and cytokine secretion, whereas lymphocytes, including T and B cells, coordinate adaptive immune responses. Several studies have shown that bioactive compounds derived from TCM can strengthen these immune functions.

Radix Bupleuri polysaccharides (RBP), isolated from a classical Chinese medicinal herb, have been shown to activate RAW264.7 macrophages. Among its fractions, acidic polysaccharides (RBP-3) exhibited stronger immunostimulatory effects by upregulating MAPK- and NF-κB-related genes through toll-like receptors TLR2 and TLR4, thereby enhancing macrophage phagocytosis and cytokine production. In cyclophosphamide-induced immunosuppressed mice, RBP also restored intestinal immune function by modulating gut microbiota composition, increasing short-chain fatty acid (SCFAs) production, and regulating CD4 + T cell differentiation, which alleviated immunosuppression (in a murine immunosuppression model) (78). These findings highlight the dual role of natural polysaccharides in innate immune activation and adaptive immune regulation.

Huaier (*Trametes robiniophila* Murr), a medicinal fungus used in TCM, exerts broad immunomodulatory effects on macrophages, dendritic cells, natural killer (NK) cells, and lymphocytes. It promotes immune cell activation, proliferation, and cytokine secretion, contributing to its immunoregulatory and antitumor activities (79, 80). Although its effects are dependent on context, this balanced immune modulation may be beneficial in infections such as calf diarrhea, where effective but controlled immune activation is required.

Dehydroandrographolide (Deh), an active compound from *Andrographis paniculata*, also modulates immune responses through NF-κB and Nrf2 signaling pathways. Deh suppresses inflammatory cytokine production and exhibits antiviral and antibacterial activities (81). This multipronged effect supports its traditional use in infectious diseases and suggests potential value in enhancing immune defenses against pathogens causing calf diarrhea.

Astragaloside IV (AS-IV), a major constituent of *Astragalus mongholicus*, enhances macrophage activity and overall immune function in immunosuppressed models. AS-IV activates the HIF-1*α*/NF-κB signaling pathway, increasing macrophage migration, phagocytosis, and the secretion of TNF-α, IL-6, and IL-1β, while reducing immunosuppressive cytokines such as IL-10 and TGF-β1. In cyclophosphamide-induced immunosuppressed mice, AS-IV restored immune organ indices, enhanced NK cell activity, and improved lymphocyte proliferation (82). These immunostimulatory effects may help strengthen host defense against infectious agents responsible for calf diarrhea.

Moreover, Rediocide-A, extracted from TCM, can potentiate NK cell-mediated cytotoxicity by increasing granzyme B and interferon-*γ* production and downregulating immune checkpoint molecules such as CD155 on target cells (83). Although this study focused on cancer, the underlying principle of enhancing lymphocyte cytotoxic function is relevant for improving immune responses in calves with diarrhea.

Collectively, these findings indicate that natural products from TCM enhance immune cell activity through multiple mechanisms, including activation of macrophage signaling pathways such as MAPK, NF-κB, and HIF-1α, modulation of cytokine profiles toward pro-inflammatory and antiviral responses, enhancement of lymphocyte proliferation and cytotoxicity, and restoration of immune homeostasis under immunosuppressed conditions. This integrated immune enhancement likely contributes to improved resistance to pathogens responsible for calf diarrhea. Combining these natural immunomodulators could provide a promising strategy for prevention and treatment (78–83).

### 5.2 Regulation of immune factor secretion

Immunoglobulins, particularly IgA and IgG, are key mediators of the humoral immune response, providing protection against pathogens by neutralizing toxins and facilitating clearance. Elevating IgA at mucosal surfaces and IgG in serum enhances calves’ resistance to enteric infections, contributing to both the prevention and treatment of diarrhea.

Traditional Chinese medicine (TCM) formulations have been shown to modulate immune responses by promoting immunoglobulin production. For example, Yigong San (YGS), a classical TCM formula for inflammatory gastrointestinal disorders, alleviated diarrhea symptoms and improved immune status in Holstein calves by modulating cytokine profiles and enhancing antioxidant capacity (12). Although cytokine suppression and oxidative stress reduction were primary endpoints, the observed immune improvements suggest an upregulation of IgA and IgG, strengthening both mucosal and systemic defense.

IgA plays a central role in neutralizing pathogens at the intestinal barrier, preventing adhesion and invasion, whereas serum IgG contributes to systemic protection by opsonizing pathogens and activating complement pathways. By enhancing both immunoglobulins, natural products and TCM formulations provide dual protection against diarrheal pathogens. These effects may partly result from restored metabolic balance and decreased pro-inflammatory cytokines (12), highlighting the interconnectedness of metabolism and immune regulation.

The enhancement of immunoglobulin secretion may also involve B-cell activation and differentiation. By reducing inflammatory mediators such as IL-1β, TNF-*α*, and IFN-*γ*, YGS creates a favorable environment for immunoglobulin synthesis. Additionally, enrichment of arginine and proline metabolism pathways observed in YGS-treated calves (12) may support immune modulation, suggesting that natural compounds improve both symptomatic outcomes and underlying immune competence through combined metabolic and immunological mechanisms.

In summary, upregulation of IgA and IgG is a pivotal mechanism by which TCM and natural products enhance mucosal and systemic immunity in calves with diarrhea. By integrating cytokine regulation, antioxidant support, and metabolic pathway modulation, these interventions provide a comprehensive approach to immune enhancement. Future studies quantifying immunoglobulin levels and B-cell activity will further clarify the mechanisms underlying these immunomodulatory effects (12).

### 5.3 Enhancing the body’s disease resistance

Enhancing the innate and adaptive immune capabilities of neonatal calves is a crucial strategy for improving their resistance to pathogens responsible for diarrhea, a leading cause of morbidity and mortality in young ruminants. The immature immune system and underdeveloped gastrointestinal barrier in newborn calves render them highly susceptible to infection by various enteric pathogens. Although effective, traditional antibiotic treatments are increasingly limited by the emergence of drug-resistant bacteria and concerns about antibiotic residues, underscoring the need for alternative interventions that can bolster the calf’s defense mechanisms (1, 10). Natural products, including Chinese herbal medicines and probiotics, have gained attention due to their immunomodulatory properties and minimal side effects.

Several studies have demonstrated that certain traditional Chinese medicine formulations can enhance the antioxidant capacity and regulate immune responses in diarrheic calves. For instance, Yigong San, a classic herbal formula, was shown to significantly elevate serum antioxidant enzymes such as catalase, glutathione peroxidase, and superoxide dismutase, while concurrently reducing malondialdehyde, a marker of oxidative stress. Moreover, YGS suppressed proinflammatory cytokines, including IL-1*α*, TNF-α, IL-1β, and IFN-*γ*, which are typically elevated during enteric infections. This anti-inflammatory effect, coupled with the modulation of arginine and proline metabolism pathways, suggests that YGS supports immune homeostasis and reduces intestinal inflammation, thereby enhancing the calf’s ability to resist pathogenic insults (12).

Probiotics are another promising avenue for enhancing disease resistance in calves. Specific strains, such as *Lactiplantibacillus plantarum* HOKKAIDO (Lp-HKD) and *Pediococcus pentosaceus* SNF15, have been shown to activate bovine immune cells through Toll-like receptor (TLR) signaling pathways, leading to increased production of key cytokines, such as IL-1β, IL-6, IL-10, and IFN-*γ*. These cytokines orchestrate both innate and adaptive immune responses, promoting pathogen clearance and tissue repair. Notably, Lp-HKD stimulation of peripheral blood mononuclear cells (PBMCs) enhanced antiviral cytokine production and exhibited protective effects against bovine rotavirus *in vitro*, indicating its potential for preventing viral diarrhea (84). Similarly, *P. pentosaceus* SNF15 improved intestinal mucosal integrity by upregulating tight junction proteins (occludin, claudin, and ZO-1) and mucin (MUC2) while reducing proinflammatory cytokines in a murine model of *E. coli* K99-induced diarrhea, collectively strengthening the intestinal barrier and modulating immune responses against enteric pathogens (14).

Micronutrient supplementation, particularly with zinc, plays a pivotal role in supporting calf immunity. Zinc is essential for maintaining epithelial barrier function and modulating immune cell activity. Evidence indicates that Zn supplementation enhances immunoglobulin production, especially IgG and IgM, which are critical for humoral immunity against pathogens. Additionally, Zn reduces proinflammatory cytokine production, which may alleviate inflammation-associated anorexia and contribute to faster recovery from diarrhea. However, the effects of Zn on specific innate immune cell functions remain inconclusive, highlighting the need for further research to optimize dosing and formulations for maximal immunological benefit (77).

Natural prebiotics, such as *Saccharomyces boulardii* cell wall polysaccharides, have demonstrated immunomodulatory effects that translate into enhanced growth performance and reduced diarrhea incidence in newborn calves. Supplementation with 500 mg/day SBWP increased serum IgG and anti-inflammatory cytokine IL-10 levels while decreasing proinflammatory cytokines IL-1, IL-6, and TNF-*α*, suggesting a shift toward an anti-inflammatory state conducive to intestinal healing. SBWP also modulated the intestinal microbiota by increasing the abundance of beneficial bacteria, such as Lactobacillus and Bifidobacterium, and suppressing pathogenic *Escherichia coli* and *Clostridium perfringens* populations, thereby reducing the incidence of diarrhea in calves (45).

Genetic factors also influence disease resistance in calves, as evidenced by studies on Holstein populations exposed to prenatal heat stress. Maternal genetic effects associated with immune response pathways, such as innate immunity and protein ubiquitination, are linked to susceptibility to diarrhea and pneumonia. The identification of candidate genes involved in the negative regulation of viral life cycles and immune modulation provides a genetic basis for breeding strategies aimed at enhancing disease resistance (85).

Collectively, these findings underscore the potential of natural products, probiotics, micronutrients, and genetic selection to enhance disease resistance in neonatal calves. Continued research integrating immunological, microbiological, and genetic perspectives is essential for developing effective and sustainable strategies that reduce reliance on antibiotics while improving calf health outcomes (77, 84, 85).

## 6 Effects of traditional Chinese medicine and natural products on gut microecology

### 6.1 Modulation of intestinal microbiota composition

Intestinal microbiota plays a pivotal role in maintaining gut health and resisting pathogenic infections, especially in neonatal calves vulnerable to diarrhea caused by bacterial pathogens such as enteroaggregative *Escherichia coli* (EAEC). Natural compounds derived from medicinal plants, such as Gallic acid, have demonstrated significant potential in modulating the gut microbial community to promote beneficial bacteria and suppress harmful species. Recent studies have shown that GA is strongly correlated with commensal bacteria such as *Collinsella* and *Coriobacterium*, which are key microbial markers associated with colonization resistance in neonatal calves (67). These commensals contribute to the maintenance of intestinal homeostasis by competing with pathogenic bacteria and producing host-beneficial metabolites.

Moreover, GA administration has been shown to increase the production of short-chain fatty acids, which are important metabolites derived from the fermentation of dietary fibers by beneficial gut microbes. SCFAs, such as butyrate, propionate, and acetate, serve as energy sources for colonocytes, strengthen the intestinal barrier, and exert anti-inflammatory effects. The positive correlation between GA, SCFAs, and the abundance of beneficial bacteria suggests that GA not only directly inhibits pathogenic bacteria but also indirectly fosters a gut environment conducive to probiotic proliferation (67). This dual action is crucial in neonatal calves, in which the immature immune systems and gut microbiota are susceptible to disruption by pathogens.

Experimental evidence from neonatal mouse models further supports the role of GA in modulating the gut microbiota structure. Oral administration of GA resulted in the enrichment of bacterial families such as Clostridia_UCG-014, *Lachnospiraceae*, *Oscillospiraceae*, and *Enterococcaceae*, all of which are known to play beneficial roles in gut health and SCFA production (67). These changes in microbial composition were associated with the attenuation of colitis symptoms induced by ESBL-producing EAEC infection, highlighting the therapeutic potential of GA in mitigating intestinal inflammation through microbiota modulation. Additionally, fecal microbiota transplantation (FMT) from GA-treated animals conferred enhanced protection against bacterial infection, demonstrating the importance of a balanced microbial community in conferring disease resistance.

The ability of GA to directly inhibit bacterial growth and reduce cell adherence of pathogenic EAEC strains also contributes to its protective effects. By limiting pathogen colonization and simultaneously promoting beneficial microbes, GA creates a competitive environment that is unfavorable to harmful bacteria. This mechanism reduces the severity of diarrhea and suggests its potential as an antibiotic alternative, although its effects on multidrug-resistant bacterial emergence require further study (67). Consequently, GA and similar natural products represent promising alternatives or adjuncts to conventional antimicrobial therapies for the prevention and treatment of calf diarrhea.

Taken together, these findings underscore the significance of natural compounds, such as Gallic acid, in reshaping the gut microbiota to enhance beneficial bacterial populations and suppress pathogens. This modulation of the intestinal ecosystem is a key strategy for preventing and managing neonatal calf diarrhea, providing a foundation for developing safer and more effective therapeutic interventions. Future research should explore the specific interactions between GA, gut microbiota, and host immune responses across diverse calf populations and environmental conditions to optimize clinical applications.

### 6.2 Improvement of intestinal environment

The intestinal environment plays a pivotal role in maintaining the health and growth of neonatal calves, particularly in preventing and treating diarrhea. One of the critical aspects of intestinal health is the production of short-chain fatty acids (SCFAs), which are metabolites generated primarily through the fermentation of dietary fibers and polysaccharides by the gut microbiota. SCFAs, including acetate, propionate, and butyrate, contribute to gut health and microbial balance, although their roles in acid–base balance and direct inhibition of pathogens require further elucidation (86, 87).

Traditional Chinese Medicine and natural products have demonstrated considerable efficacy in modulating gut microbiota composition to enhance SCFA production. Polysaccharides derived from various TCM herbs serve as prebiotic substrates for gut bacteria, promoting the growth of SCFA-producing genera, such as *Lachnospiraceae* and *Clostridia* (67, 87). For instance, Gallic acid, a plant-derived phenolic compound, has been shown to enrich commensal bacteria such as Clostridia_UCG-014 and *Lachnospiraceae* in neonatal mice, leading to increased SCFA levels and amelioration of colitis symptoms (67). This suggests that TCM components can restore intestinal homeostasis by fostering a microbial environment conducive to SCFA synthesis, which, in turn, supports the integrity of the intestinal barrier and modulates immune responses.

SCFAs are associated with gut health and may influence colonization by pathogens such as extended-spectrum *β*-lactamase-producing enteroaggregative *Escherichia coli*, a common causative agent of calf diarrhea (67). The maintenance of a slightly acidic environment through SCFA accumulation inhibits pathogen colonization and enhances nutrient absorption and mucosal healing. TCM formulations and natural products, indirectly reduce the incidence and severity of diarrhea in calves by promoting SCFA production.

**Figure 4:**
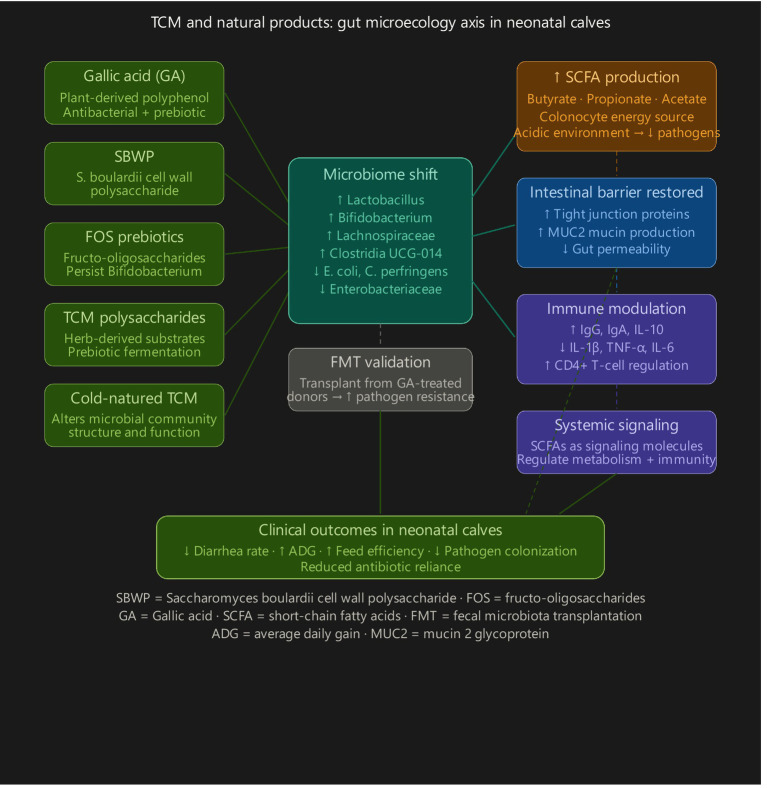
TCM and natural products: gut microecology axis in neonatal calves.

The interplay between TCM and gut microbiota extends to the modulation of metabolic pathways associated with SCFA biosynthesis. Studies have demonstrated that cold-natured TCMs significantly alter the gut microbial community structure and functional repertoire, including those involved in nutrient absorption (88). Such modulation reflects the multi-target nature of TCM, where the enhancement of SCFA generation is one of several mechanisms contributing to improved intestinal health of the host. This multifaceted regulation underscores the potential of TCM and natural products as alternatives or adjuncts to antibiotics in managing calf diarrhea by restoring the gut microbial balance and enhancing beneficial metabolite production (Figure 4).

Additionally, the benefits of increased SCFA production extend beyond the local gut effects. SCFAs serve as signaling molecules that influence systemic immune modulation and metabolic regulation (89). This systemic impact highlights the importance of maintaining a healthy gut environment not only for gastrointestinal function but also for broader physiological homeostasis int. the body.

In summary, the application of TCM and natural products in calf diarrhea management involves enhancing SCFA production to maintain intestinal acid–base balance, inhibit pathogenic bacteria, and support mucosal integrity in calves. These effects are mediated by the modulation of gut microbiota composition and metabolic activity, offering a promising avenue for improving intestinal health and reducing the disease burden in neonatal calves. Future research should focus on elucidating the mechanisms of SCFA synthesis promotion and therapeutic strategies for calf diarrhea prevention and treatment (67, 86, 87).

### 6.3 Promotion of intestinal barrier function recovery

The integrity and proper function of the intestinal barrier are critical in preventing and managing calf diarrhea, as disruption of this barrier often leads to increased intestinal permeability, facilitating the translocation of pathogens and toxins that exacerbate the severity of the disease. One of the key mechanisms for restoring and strengthening the intestinal barrier involves the upregulation of tight junction proteins, which form a physical seal between adjacent epithelial cells, thereby reducing intestinal permeability and maintaining mucosal homeostasis. Traditional Chinese medicine formulations, such as Yigong San, have shown promising effects in this regard, providing a potential alternative to conventional antibiotic therapies, which often carry the risks of residue and resistance.

Recent studies investigating the therapeutic effects of YGS in diarrheic calves have demonstrated its capacity to improve intestinal barrier integrity by modulating the molecular and biochemical pathways related to oxidative stress and inflammation. In a controlled study involving Holstein calves with natural diarrhea, treatment with YGS for 7 days resulted in significant alleviation of diarrheal symptoms, alongside improvements in biochemical markers indicative of intestinal health. Importantly, YGS administration enhanced the activity of antioxidant enzymes, such as catalase, glutathione peroxidase, and superoxide dismutase, while concurrently reducing malondialdehyde levels, a marker of lipid peroxidation and oxidative damage. This antioxidative effect is crucial in mitigating oxidative stress implicated in disease, although specific effects on tight junction integrity and signaling pathways require further evidence (12).

Concomitantly, YGS suppressed the expression of multiple proinflammatory cytokines, including IL-1*α*, TNF-α, IL-1β, IL-17A, IL-18, IL-21, and IFN-*γ*, as well as chemokines such as CXCL9 and CXCL10, which are known to contribute to intestinal inflammation and barrier dysfunction. The reduction in these inflammatory mediators likely contributes to the restoration of tight junction proteins by decreasing epithelial cell apoptosis and promoting mucosal healing. This dual action of antioxidative enhancement and inflammation suppression creates a favorable microenvironment for the recovery of intestinal barrier function. YGS’s modulation of immune function by YGS may contribute to improved intestinal health, although its direct effects on tight junction proteins and barrier integrity remain to be demonstrated (12).

Moreover, metabolomic profiling revealed that YGS treatment corrected the metabolic disturbances associated with diarrhea, particularly enriching pathways involved in arginine and proline metabolism. Metabolites such as 1-methylhydantoin and ornithine were found to be negatively correlated with proinflammatory cytokine levels, suggesting that metabolic regulation by YGS may play a role in mitigating inflammation and promoting mucosal repair. Since arginine is a known precursor for the synthesis of nitric oxide, which modulates vascular tone and immune responses, its enhanced metabolism may contribute to improved mucosal blood flow and epithelial regeneration. This metabolic adjustment may support intestinal health, although direct evidence for the maintenance and restoration of tight junction protein expression is lacking (12).

In summary, the application of YGS in treating calf diarrhea exemplifies how traditional herbal formulations can promote intestinal barrier function recovery by strengthening tight junction protein expression and reducing intestinal permeability. This is achieved through the synergistic effects of upregulating antioxidative enzymes, suppressing proinflammatory cytokines, and correcting metabolic imbalances. These findings underscore the potential of YGS as a safe and effective alternative to antibiotics for managing calf diarrhea, with the added benefit of enhancing mucosal barrier integrity to prevent recurrence and promote long-term intestinal health in calves. Further research into the specific tight junction proteins affected by YGS and the signaling pathways involved would deepen our understanding and facilitate the optimization of such natural therapies.

## 7 Safety and toxicological evaluation of traditional Chinese medicine and natural products

### 7.1 Toxicity and side effect evaluation

The evaluation of toxicity and side effects is a critical aspect in the application of traditional Chinese medicine and natural products for the prevention and treatment of calf diarrhea. Acute and chronic toxicity studies are essential to ensure the safety of these agents, especially considering their potential widespread use in livestock. In recent research focusing on Yigong San, a traditional Chinese medicine formula, no overt signs of acute toxicity were observed in treated calves during the seven-day administration period. The study incorporated Biochemical analyses of serum samples revealed that YGS alleviated diarrhea symptoms and improved abnormal biochemical indicators (12). This suggests that YGS has a favorable safety profile in the short term, although further chronic toxicity studies are warranted to confirm its long-term safety.

YGS treatment had a protective effect on hepatic and renal functions, which are key indicators of systemic toxicity. Biochemical parameters related to liver and kidney function showed improvement post-treatment, aligning with enhanced antioxidant enzyme activities, such as catalase, glutathione peroxidase, and superoxide dismutase. These enzymes play crucial roles in mitigating oxidative stress and contribute to improved antioxidant capacity (12). The reduction in malondialdehyde levels further supports the notion that YGS reduces lipid peroxidation, thereby potentially protecting the hepatocytes and renal cells from oxidative injury. These findings imply that YGS does not exert hepatotoxic or nephrotoxic effects; rather, it may contribute to the restoration of normal liver and kidney functions in diarrheic calves.

Moreover, the immunomodulatory effects of YGS, characterized by the suppression of proinflammatory cytokines such as IL-1*α*, TNF-α, IL-1β, and IFN-*γ*, indicate systemic anti-inflammatory action (12). This anti-inflammatory profile may help mitigate secondary damage to the liver and kidneys, which often occurs in severe or prolonged diarrheal diseases. Metabolomic analysis revealed enrichment in arginine and proline metabolism pathways, with metabolites such as 1-methylhydantoin and ornithine negatively correlating with inflammatory cytokine levels, further suggesting a metabolic environment conducive to tissue repair and reduced toxicity.

While these results are promising, it is important to note that the current evidence primarily stems from a relatively short treatment duration and limited sample size. The absence of reported adverse effects in this context supports the potential of YGS as a safe alternative to antibiotics for treating calf diarrhea. However, comprehensive chronic toxicity studies, including histopathological examinations of the liver and kidney tissues, are necessary to fully ascertain the long-term safety profiles of YGS and other natural products in veterinary applications. Additionally, variability in individual responses and potential interactions with other medications or feed additives should be carefully evaluated in future studies.

In summary, YGS demonstrates a beneficial safety profile with no significant toxic effects on hepatic and renal functions during the treatment of calf diarrhea, as supported by improved antioxidant status and reduced inflammatory markers. This highlights its potential as a safe and effective alternative to conventional antibiotics, with the added advantage of modulating immune and metabolic pathways to support organ health (12). Further investigations are recommended to validate these findings over extended periods and in larger cohorts to ensure the broader applicability and safety of these natural therapeutic agents in livestock management.

### 7.2 Safety analysis of dosage and administration routes

The oral administration of traditional Chinese medicine formulations and natural products for the prevention and treatment of calf diarrhea necessitates careful evaluation of dosage ranges and dosing frequency to ensure their safety and therapeutic efficacy. In recent research investigating Yigong San, a TCM formula traditionally used for inflammatory gastrointestinal disorders, oral dosing was applied to diarrheic calves over a seven-day treatment course. This study involved 20 diarrheic Holstein calves receiving YGS, and they were compared with untreated diarrheic and healthy control groups, providing a controlled framework to assess both efficacy and safety parameters (12).

The oral dosage regimen of YGS used in this study was effective in alleviating diarrheal symptoms without observable adverse effects, underscoring its safety profile at the administered dose and frequency. Serum biochemical parameters measured post-treatment indicated improvements in liver and kidney function markers, which are critical indicators of systemic toxicity. Furthermore, oxidative stress markers such as catalase, glutathione peroxidase, and superoxide dismutase were significantly elevated, while malondialdehyde levels decreased, suggesting that the administered dose did not induce oxidative damage but rather enhanced antioxidant defenses. These findings collectively support the notion that oral dosing of YGS within the tested range is safe and may confer protective effects against oxidative stress in calves with diarrhea (12).

In terms of dosing frequency, daily oral administration over seven consecutive days was sufficient to achieve therapeutic effects without toxic accumulation or adverse immunological reactions. The suppression of multiple proinflammatory cytokines, including IL-1*α*, TNF-α, IL-1β, IL-17A, IL-18, IL-21, IFN-*γ*, and chemokines CXCL9 and CXCL10, indicates that YGS modulates immune responses in a manner that is both effective and safe at the given dosage schedule. This immunomodulatory effect suggests balanced immune regulation, although evidence regarding the absence of overt immunosuppression is not explicit (12).

Metabolomic profiling further demonstrated that the administered dose of YGS positively influenced metabolic pathways, notably enriching arginine and proline metabolism, which are important for immune functions and tissue repair. The negative correlation between metabolites such as 1-methylhydantoin and ornithine with pro-inflammatory cytokine levels reinforces the safety of the dosing regimen by indicating a systemic shift towards resolving inflammation and restoring metabolic homeostasis. Such metabolic modulation has been observed, although data on metabolic toxicity or dosage appropriateness have not been explicitly reported (12).

Taken together, these data suggest that oral administration of YGS at the tested dosage and frequency is safe for calves suffering from diarrhea, with additional benefits in antioxidant capacity and immune function regulation. These results provide a foundation for optimizing the dosing strategies of TCM and natural products for veterinary applications. Future studies should explore dose escalation or alternative administration routes to further refine safety margins and maximize therapeutic outcomes, particularly considering variations in calf age, severity of diarrhea, and environmental factors.

### 7.3 Drug interactions and risk of resistance

When considering the use of traditional Chinese medicine formulas and natural products in the prevention and treatment of calf diarrhea, it is essential to address potential drug interactions and the risk of developing resistance, especially when these agents are used in combination with conventional drugs. The combination of herbal formulations, such as Yigong San, with antibiotics or other drugs requires careful evaluation to avoid adverse interactions that could compromise therapeutic efficacy or animal safety. YGS, a traditional Chinese medicine formula, has been shown to effectively alleviate diarrhea by enhancing the antioxidant capacity and modulating immune responses, thereby offering a promising alternative to antibiotics (12). However, the concurrent use of YGS with antibiotics can lead to complex pharmacodynamic and pharmacokinetic interactions that may alter drug metabolism or immune modulation. For instance, the antioxidant and anti-inflammatory effects of YGS may influence the metabolism of co-administered drugs metabolized through oxidative pathways, potentially affecting their plasma concentrations and therapeutic windows.

Furthermore, the risk of drug-resistance development remains a critical concern in calf diarrhea management. Antibiotic use is a well-known driver of antimicrobial resistance, which poses significant challenges to both animal and public health. Natural products, such as YGS may help mitigate this risk by reducing the reliance on antibiotics. A study on YGS demonstrated its capacity to suppress multiple proinflammatory cytokines, such as IL-1*α*, TNF-α, and IFN-*γ*, and to regulate immune function without the direct use of antibiotics, suggesting a lower propensity for inducing resistance (12). This immunomodulatory mechanism could provide a dual benefit by controlling infection and reducing inflammation, thereby reducing the selective pressure for resistant bacterial strains. Nevertheless, the possibility that herbal compounds might interact with antibiotics to either potentiate or inhibit their activity warrants further pharmacological studies to delineate safe and effective combination regimens.

In practical applications, when integrating TCM or natural products with existing drug protocols, veterinarians should consider the timing, dosage, and administration sequence to minimize adverse interactions. For example, staggered dosing may reduce competition for metabolic enzymes or transporters, thereby preserving the activity of both agents. Additionally, monitoring biochemical and immune parameters during combined therapy can provide early indicators of potential interactions or toxicities. Metabolomic analyses of calves treated with YGS revealed improvements in arginine and proline metabolism pathways and correlations between certain metabolites and inflammatory cytokine levels (12). These metabolic shifts underscore the importance of understanding the broader physiological effects of natural products on drug metabolism and immune responses.

Moreover, the use of natural products as antibiotic alternatives may also impact the gut microbiota composition, which plays a crucial role in drug metabolism and resistance. While YGS has shown beneficial effects in improving calf health, its influence on the microbiome and subsequent drug interactions remains to be fully elucidated. By promoting a healthier gut environment and reducing inflammation, YGS could indirectly reduce the colonization and proliferation of resistant pathogens, thereby contributing to resistance management strategies. This highlights the potential for integrating TCM with conventional therapies not only to treat symptoms but also to address the underlying causes of disease and resistance emergence.

In summary, the combined use of herbal medicines, such as YGS, with antibiotics or other drugs for calf diarrhea requires a comprehensive understanding of possible drug interactions and resistance risks. While YGS shows promise as a safe and effective alternative that modulates immune and metabolic pathways, careful consideration of pharmacological interactions and vigilant monitoring are necessary to optimize therapeutic outcomes and minimize adverse effects. Future research should focus on detailed pharmacokinetic studies and clinical trials to establish evidence-based guidelines for the co-administration of natural products and conventional drugs in veterinary practices.

## 8 Clinical application and case analysis of traditional Chinese medicine and natural products in the prevention and treatment of calf diarrhea

### 8.1 Typical traditional Chinese medicine formulations and their clinical efficacy evaluation

The clinical efficacy of typical traditional Chinese medicine formulations in the treatment of calf diarrhea has been increasingly validated through randomized controlled trials and biochemical assessments, highlighting their potential as effective alternatives to conventional antibiotics. One representative formulation, Cangpu Oral Liquid, derived from a traditional herbal formula historically used for gastrointestinal diseases in animals, was evaluated in a randomized controlled field trial involving 246 diarrheal Holstein calves aged 2 to 15 days. The study demonstrated that 101 out of 123 calves treated with CP recovered from diarrhea, compared to 77 out of 123 in the apramycin antibiotic group. Notably, the CP group exhibited a significantly higher average daily gain (ADG) of 211.45 grams/day versus 164.56 grams/day in the antibiotic group (*p* = 0.001), and a shorter recovery time from diarrhea (3.90 days versus 6.62 days, *p* = 0.001), indicating not only symptomatic relief but also improved growth performance post-treatment. These findings suggest that CP may offer superior clinical benefits for neonatal calf diarrhea with fewer concerns related to antibiotic resistance and residues (10). The enhanced growth metrics observed also imply that CP may support nutrient absorption or gut health, beyond mere symptom control.

Another notable TCM formula, Yigong San, traditionally prescribed for inflammatory gastrointestinal disorders, has been investigated for its therapeutic effects on calf diarrhea through a controlled study involving healthy controls, natural diarrhea calves, and YGS-treated calves. YGS treatment significantly alleviated diarrhea symptoms and corrected abnormal serum biochemical markers. Importantly, YGS enhanced the antioxidant defense system by increasing the serum levels of catalase, glutathione peroxidase, and superoxide dismutase, while concurrently reducing the levels of malondialdehyde, a marker of oxidative stress. This antioxidant enhancement was accompanied by the suppression of multiple proinflammatory cytokines, such as IL-1*α*, TNF-α, IL-1β, IL-17A, IL-18, IL-21, IFN-*γ*, and chemokines CXCL9 and CXCL10. Metabolomic profiling further revealed that YGS modulated metabolic pathways, particularly arginine and proline metabolism, with metabolites, such as 1-methylhydantoin and ornithine showing negative correlations with pro-inflammatory cytokine levels. These results collectively indicate that YGS exerts multifaceted therapeutic effects by improving antioxidant capacity, reducing inflammation, and regulating immune responses, which may contribute to its efficacy in treating calf diarrhea (12). These mechanistic insights support the potential of YGS as a safe and effective antibiotic alternative, emphasizing the importance of metabolic and immune modulation in disease resolution.

In addition to herbal formulations, micronutrient supplementation, particularly zinc, has been recognized for its adjunctive role in enhancing calf immunity and mitigating diarrhea severity. A scoping review examining the impact of Zn on calf immunity revealed moderate evidence supporting the benefits of Zn supplementation in maintaining epithelial barriers of integumental and mucosal surfaces, which are critical in preventing pathogen invasion during diarrheal episodes. Zn was also associated with increased immunoglobulin production, especially IgG and IgM, and reduced proinflammatory cytokine production, potentially decreasing inflammation-associated hypophagia and shortening the duration of clinical signs in diarrheic calves. However, the review highlighted insufficient evidence regarding the effects of Zn on innate immune cells and T-cell functions, underscoring the need for further research to optimize Zn dosing and formulations for maximal therapeutic benefit (77). The role of Zn in reinforcing mucosal immunity may complement the actions of TCM formulations, suggesting a potential synergistic approach to managing calf diarrhea.

**Figure 5:**
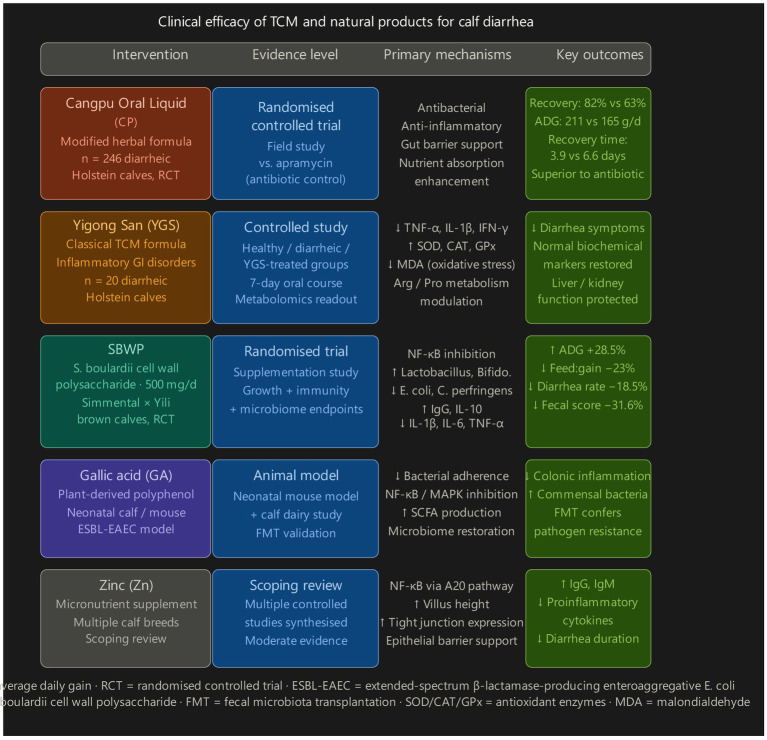
Clinical efficacy of TCM and natural products for calf diarrhea.

Collectively, these clinical evaluations of typical TCM formulations and micronutrient supplementation demonstrate promising efficacy in the prevention and treatment of calf diarrhea. The observed benefits extend beyond symptom relief and include growth promotion, antioxidant enhancement, inflammatory modulation, and immune regulation. These multifactorial effects align with the holistic principles of TCM and provide a scientific basis for its integration into calf health management strategies. Future studies should focus on large-scale clinical trials, standardized dosing regimens, and mechanistic exploration to further validate and optimize these treatments for broader applications in the dairy industry (Figure 5).

### 8.2 Application examples of natural product monomers and compound formulations

The use of natural product monomers and compound formulations in the prevention and treatment of calf diarrhea has garnered increasing attention because of their multifaceted therapeutic effects and potential to serve as alternatives to antibiotics. One notable example is Yigong San, a traditional Chinese medicine formula historically used for inflammatory gastrointestinal disorders. A controlled study investigating YGS in diarrheic Holstein calves demonstrated that YGS administration effectively alleviated diarrheal symptoms and improved abnormal biochemical indices. Specifically, YGS enhanced the activities of antioxidant enzymes, including catalase, glutathione peroxidase, and superoxide dismutase, while reducing malondialdehyde levels, a marker of lipid peroxidation. Furthermore, YGS suppressed proinflammatory cytokines, such as IL-1*α*, TNF-α, IL-1β, and IFN-*γ*, indicating potent anti-inflammatory properties. Metabolomic profiling revealed that YGS modulates arginine and proline metabolism pathways, with metabolites such as 1-methylhydantoin and ornithine negatively correlated with inflammatory cytokine levels, suggesting a mechanistic link between metabolic regulation and immune modulation (12). These findings support the therapeutic potential of YGS as a safe and effective antibiotic alternative for calf diarrhea management.

Another compelling example is the supplementation of *Saccharomyces boulardii* cell wall polysaccharide, a natural prebiotic, which has been shown to enhance growth performance and reduce diarrhea incidence in newborn calves. In a randomized trial involving Simmental × Yili brown calves, daily supplementation with 500 mg SBWP significantly increased body weight and average daily gain by approximately 4.9% and 28.5%, respectively, while decreasing the feed-to-gain ratio by nearly 23%. Importantly, SBWP supplementation reduced fecal scores and diarrhea rates by 31.6% and 18.5%, respectively, compared with the control group. Immunologically, calves receiving SBWP exhibited elevated serum IgG and IL-10 levels, along with decreased proinflammatory cytokine IL-1, IL-6, and TNF-*α* levels. Microbiota analysis indicated a favorable shift, with reduced *Escherichia coli* and *Clostridium perfringens* populations and increased Lactobacillus and Bifidobacterium abundance. These results highlight SBWP’s role in enhancing immunity, modulating gut microbiota, and improving intestinal health, thereby contributing to diarrhea mitigation in calves (45). This evidence suggests that SBWP can be integrated into calf feeding regimens to promote health and growth while reducing reliance on antibiotics.

Passive immunization strategies employing IgY antibodies derived from hyperimmunized hens offer another innovative natural product-based intervention. A product named IgY DNT, containing IgY antibodies targeting group A rotavirus, coronavirus, enterotoxigenic *Escherichia coli*, and Salmonella species, was evaluated in newborn Holstein calves. Oral administration of IgY DNT twice daily during the first 2 weeks of life delayed diarrhea onset, reduced its severity and duration, and shortened the viral shedding period compared to untreated calves. This approach effectively complements existing preventive measures against neonatal calf diarrhea caused by viral infections and provids a biological alternative to conventional antimicrobials. The specificity of IgY DNT underscores its potential for widespread application in dairy farms for controlling enteric infections (90).

Inulin-related prebiotics, such as fructo-oligosaccharides (FOS), have also been investigated for their benefits in calf gut health. Supplementation with FOS in newborn Holstein calves promoted a time-dependent increase in ADG and short-chain fatty acid concentrations, which are vital for intestinal epithelial health and energy metabolism in the host. Additionally, FOS delayed the natural decline of beneficial Bifidobacterium species, supporting the maturation and stabilization of the hindgut microbiota. Microbiome modulation is crucial for enhancing disease resistance and nutrient absorption during the vulnerable neonatal period. These findings provide a theoretical and practical basis for incorporating FOS into early life dietary interventions to improve growth performance and reduce the incidence of diarrhea (91). The promotion of beneficial microbiota by FOS may synergize with other natural products to optimize gut health.

Gallic acid, a plant-derived polyphenol, exemplifies a natural monomer with direct antibacterial and anti-inflammatory effects against multidrug-resistant enteroaggregative *Escherichia coli* infections in neonatal calves. Studies have demonstrated that GA pre-treatment inhibits bacterial growth and adherence to intestinal epithelial cells, attenuates colonic inflammation, and restores SCFA production. Fecal microbiota transplantation from GA-treated neonatal mice further confirmed the restoration of gut microbial homeostasis, with the enrichment of beneficial taxa, such as Clostridia_UCG-014 and *Lachnospiraceae*. These changes were correlated with improved clinical outcomes and colitis remission. GA’s dual role in modulating both microbial composition and host immune responses positions it as a promising candidate for antibiotic alternatives or adjunct therapies in managing ESBL-EAEC-induced calf diarrhea (67). The integration of such natural compounds can mitigate the spread of antimicrobial resistance in livestock.

Collectively, these application examples illustrate that natural product monomers and compound formulations exert therapeutic effects through diverse mechanisms, including immune modulation, antioxidant enhancement, microbiota regulation, and direct antimicrobial activity. Their multifactorial actions not only alleviate clinical symptoms but also address the underlying pathophysiological processes of calf diarrhea. The evidence supports the continued exploration and optimization of these natural interventions to develop effective, sustainable, and safe alternatives to antibiotics for calf diarrhea prevention and treatment.

### 8.3 Issues in application and strategies for resolution

The application of traditional Chinese medicine and natural products in the prevention and treatment of calf diarrhea faces several significant challenges, the foremost of among which are quality control and dosage standardization. Unlike conventional antibiotics, which benefit from well-established manufacturing and regulatory frameworks to ensure consistent potency and purity, herbal medicines often exhibit variability in active ingredient concentrations due to differences in plant sources, harvesting times, and processing methods. This variability complicates the establishment of standardized dosages, which is critical for achieving reproducible therapeutic effects and ensuring safety. For example, a clinical trial of Cangpu Oral Liquid, a formula derived from traditional Chinese herbal medicine, demonstrated superior efficacy compared to apramycin in treating neonatal calf diarrhea, with higher recovery rates and improved average daily gain (10). However, such promising results need to be underpinned by rigorous quality control protocols to ensure batch-to-batch consistency, which remains a current limitation in the field.

Another challenge is the lack of standardized dosing regimens for herbal formulation. The pharmacokinetics and pharmacodynamics of many active compounds in TCM have not been fully elucidated, leading to empirical dosing that may vary widely between practitioners and studies. This issue is compounded by the complex multi-component nature of herbal formulas, where synergistic or antagonistic interactions among constituents can influence their efficacy and safety. For instance, Yigong San extract has shown beneficial effects in modulating antioxidant status, inflammatory cytokines, and metabolic profiles in diarrheic calves, indicating its potential as an alternative to antibiotic (12). However, the precise dosing parameters to maximize these benefits without adverse effects remain standardized. Without such standardization, it is difficult to compare outcomes across studies or to broadly implement these treatments in clinical practice.

The complexity of the gut microbiome and its role in calf health further complicate the application of natural products. Recent studies have shown that supplementation with prebiotics, such as fructo-oligosaccharides and probiotics, such as *Saccharomyces boulardii* cell wall polysaccharides, can beneficially modulate the intestinal microbiota and reduce diarrhea incidence (45, 91). However, the interactions between these supplements and herbal compounds are not well understood, raising concerns about potential variability in therapeutic outcomes. Moreover, the stability of herbal compounds during storage and upon administration, as well as their bioavailability in the gastrointestinal tract, remain underexplored areas that impact efficacy.

Several strategies have been proposed to address these challenges. First, establishing stringent quality control measures using modern analytical techniques, such as high-performance liquid chromatography (HPLC) and mass spectrometry, can ensure the identification and quantification of bioactive components in herbal products. This approach will facilitate the development of pharmacopeial standards that are specific to veterinary herbal medicines. Second, conducting dose–response studies in controlled clinical trials are essential to define optimal dosing regimens. For example, the dose-dependent effects of SBWP supplementation on growth performance and immune parameters in calves highlight the importance of precise dosing to achieve maximal benefits (45). Third, integrating multi-omics approaches, including metabolomics and microbiome profiling, can elucidate the mechanisms of action of herbal medicines and their interactions with the gut microbiota, enabling more their targeted and effective use.

Furthermore, using standardized extracts or purified active compounds instead of crude herbal mixtures may reduce variability and improve reproducibility. The development of formulations with enhanced bioavailability, such as nanoparticles or encapsulation technologies, can also improve therapeutic outcomes. Additionally, combination of herbal medicines with probiotics or prebiotics in synergistic formulations may potentiate their effects on gut health and immunity. However, these combinations require careful evaluation to avoid unintended interactions.

Finally, regulatory frameworks must evolve to accommodate the unique characteristics of TCM and natural products in veterinary medicine. Clear guidelines for quality standards, safety assessments, and efficacy evaluations are necessary to facilitate the acceptance and integration of these products into mainstream calf health management. Training and educating veterinarians and farmers on the appropriate use of these alternative therapies are equally important to ensure their rational application.

In summary, while TCM and natural products offer promising alternatives to antibiotics for calf diarrhea, overcoming challenges related to quality control, dosage standardization, and mechanistic understanding is imperative for their effective use. Addressing these issues through rigorous research, technological innovation, and regulatory support will pave the way for the safe and effective application of probiotics in calf health management.

### 8.4 Research gaps

Based on the comparison and analysis of limitations, we will more systematically outline future research directions. Specific research gaps include:

1. In-depth analysis of mechanisms of action: Although these natural products are known to have multiple effects, the specific molecular targets and signaling pathways remain unclear. Therefore, future research can employ multi-omics approaches (e.g., metabolomics, microbiomics) and network pharmacology to conduct more in-depth investigations into their mechanisms of action.
2. Synergistic and antagonistic effects with conventional drugs: When traditional Chinese medicine (TCM) is used in combination with antibiotics, how to optimize the administration sequence and dosage to minimize adverse reactions or interactions while maximizing synergistic effects currently lacks systematic research. This will also be a key focus of future studies. For instance, some studies have shown that combining TCM with antibiotics can increase the sensitivity of drug-resistant bacteria to antibiotics, thereby reducing the amount of antibiotics used.
3. Clinical translation and standardization: Establishing a standardized process for TCM, from quality control to clinical application, including the development of a veterinary-specific herbal pharmacopoeia standard, is a critical bottleneck for future application. This direction has become a major research hotspot in China and represents an important pathway for introducing traditional Chinese medicine to the world.

## 9 Conclusion

**Figure 6:**
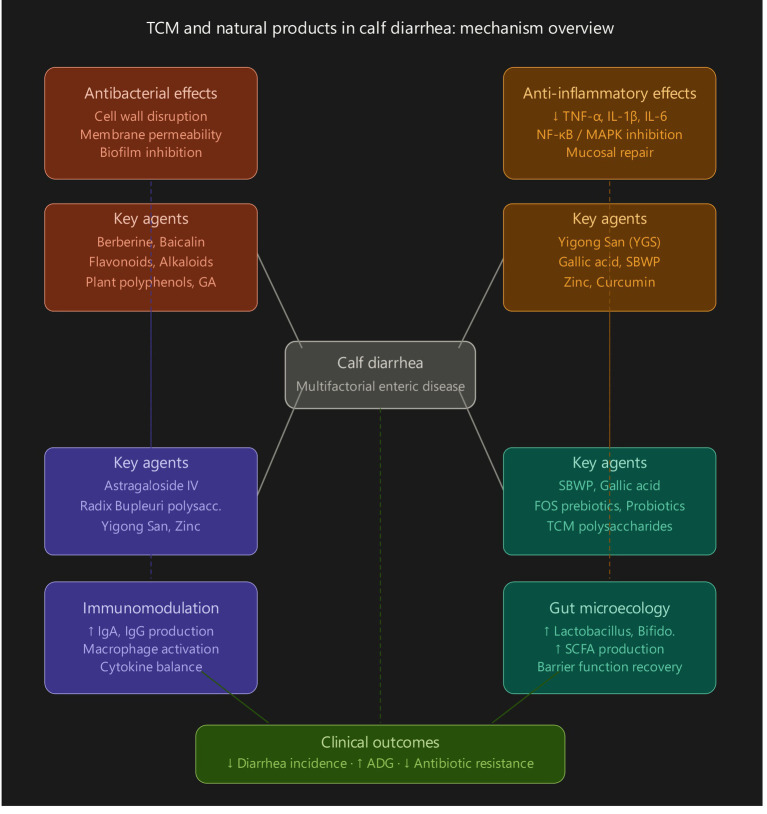
TCM and natural products in calf diarrhea: mechanism overview.

In conclusion, the multifactorial etiology of calf diarrhea necessitates a comprehensive, multi-targeted therapeutic approach to effectively manage this pervasive and economically significant condition. From an expert perspective, the integration of traditional Chinese medicine and natural products into prevention and treatment strategies offers a promising avenue that aligns well with the complex pathophysiology of calf diarrhea. The inherent multi-component and multi-target characteristics of these natural remedies provide distinct advantages by simultaneously addressing the diverse mechanisms involved, including antimicrobial, anti-inflammatory, immunomodulatory, and gut microbiota regulatory effects (For TCM mechanism overview map see Figure 6).

The evidence reviewed underscores that TCM and natural products can effectively alleviate the clinical symptoms of calf diarrhea and promote intestinal health through the synergistic modulation of multiple biological pathways. This holistic approach contrasts with the often singularly targeted pharmaceutical interventions, which may not fully address the intricate interplay of infectious agents, immune response dysregulation, and gut microbiome disturbances that are characteristic of this disorder. Moreover, the utilization of natural products aligns with the growing demand for sustainable and eco-friendly veterinary practices, reducing the reliance on conventional antibiotics and mitigating the risk of antimicrobial resistance.

Safety considerations remain paramount, as highlighted by current research indicating that when administered with appropriate dosing and quality control, TCM and natural products are generally well tolerated in calves. Nonetheless, the variability in formulations, active compound concentrations, and preparation standards necessitates rigorous standardization protocols and comprehensive toxicological assessments to ensure their consistent efficacy and safety. This is especially critical given the potential for adverse effects arising from improper dosing or contamination, which could undermine the benefits and acceptance of these treatments.

Clinical application data have demonstrated encouraging outcomes, with real-world case studies supporting the feasibility and effectiveness of incorporating TCM and natural products into routine calf management practices. These findings advocate for broader adoption; however, they simultaneously emphasize the urgent need for large-scale randomized controlled trials to robustly validate efficacy, delineate precise mechanisms of action, and optimize treatment regimens. Such research endeavors will be instrumental in overcoming the current limitations related to sample size, heterogeneity of study designs, and mechanistic ambiguity.

Balancing diverse research perspectives, it is evident that while conventional pharmacotherapy remains indispensable, the adjunctive or alternative use of TCM and natural products represents an innovative paradigm shift toward a more holistic and sustainable animal health management. Future research should prioritize integrative approaches that combine molecular, microbiological, immunological, and pharmacological methodologies to deepen our mechanistic understanding and refine therapeutic protocols. Additionally, policy frameworks and veterinary guidelines must evolve to incorporate evidence-based recommendations for these natural interventions, fostering their standardized and responsible use in the livestock industry.

Beyond their therapeutic merits, TCM formulations and natural products offer meaningful economic advantages over conventional antibiotics. By shortening recovery time, improving growth performance, and reducing the risks of drug residues and antimicrobial resistance, these alternatives address both the immediate and long-term costs associated with calf diarrhea management, supporting their integration as sustainable and economically sound options in modern animal husbandry.

Overall, TCM and natural products offer a vital green alternative for controlling calf diarrhea, with the potential to enhance animal welfare, reduce economic losses, and contribute to global efforts to combat antimicrobial resistance. Sustained investment in both foundational and translational research is essential to fully harness their capabilities and facilitate their widespread and standardized application in veterinary medicine. By embracing this multifaceted strategy, the field can move toward more effective, safe, and sustainable management of calf diarrhea, ultimately improving outcomes for animal health and agricultural productivity.
